# Advances in Technologies for Highly Active Omega-3 Fatty Acids from Krill Oil: Clinical Applications

**DOI:** 10.3390/md19060306

**Published:** 2021-05-26

**Authors:** Alessandro Colletti, Giancarlo Cravotto, Valentina Citi, Alma Martelli, Lara Testai, Arrigo F. G. Cicero

**Affiliations:** 1 Department of Drug Science and Technology, University of Turin, Via P. Giuria 9, 10125 Turin, Italy; Alessandro.colletti@unito.it (A.C.); giancarlo.cravotto@unito.it (G.C.); 2 World-Class Research Center “Digital Biodesign and Personalized Healthcare”, Sechenov First Moscow State Medical University, 8 Trubetskaya ul, 101000 Moscow, Russia; 3 Department of Pharmacy, University of Pisa, 56121 Pisa, Italy; valentina.citi@unipi.it (V.C.); alma.martelli@unipi.it (A.M.); lara.testai@unipi.it (L.T.); 4 Interdepartmental Research Centre “Nutraceuticals and Food for Health (NUTRAFOOD)”, University of Pisa, 56121 Pisa, Italy; 5 Interdepartmental Research Centre of Ageing, Biology and Pathology, University of Pisa, 56121 Pisa, Italy; 6 Medical and Surgical Sciences Department, Alma Mater Studiorum University of Bologna, 40138 Bologna, Italy; 7 IRCCS Policlinico S. Orsola-Malpighi, 40138 Bologna, Italy

**Keywords:** krill oil, omega-3 polyunsaturated fatty acids, bioavailability, nutraceuticals, dietary supplements

## Abstract

*Euphausia superba*, commonly known as krill, is a small marine crustacean from the Antarctic Ocean that plays an important role in the marine ecosystem, serving as feed for most fish. It is a known source of highly bioavailable omega-3 polyunsaturated fatty acids (eicosapentaenoic acid and docosahexaenoic acid). In preclinical studies, krill oil showed metabolic, anti-inflammatory, neuroprotective and chemo preventive effects, while in clinical trials it showed significant metabolic, vascular and ergogenic actions. Solvent extraction is the most conventional method to obtain krill oil. However, different solvents must be used to extract all lipids from krill because of the diversity of the polarities of the lipid compounds in the biomass. This review aims to provide an overview of the chemical composition, bioavailability and bioaccessibility of krill oil, as well as the mechanisms of action, classic and non-conventional extraction techniques, health benefits and current applications of this marine crustacean.

## 1. Introduction

*Euphausia superba*, commonly known as krill, is a small marine crustacean from the Antarctic Ocean that plays an important role in the marine ecosystem, serving as feed for most fish [1]. Although measuring krill biomass is difficult, it has been estimated at approximately 379 million metric tons. The Commission for the Conservation of Antarctic Marine Living Resources (CCAMLR) has set a catch limit of 620,000 tons per year to protect the marine ecosystem [2]. Nevertheless, the annual catch is around 250,000 tons, indicating use below the established limits, which is probably due to the difficulty in conserving krill and its fragility [3].

In fact, krill is commonly used in the sport fishing market as well as in the aquaculture industry. However, in recent years, krill has been successfully investigated for its role as a nutritional supplement to improve human health. This is because krill is rich in nutrients, including vitamins A and E, minerals, n-3 polyunsaturated fatty acids (n-3 PUFAs), phospholipids (PLs), astaxanthin and flavonoids [4].

Particular attention has been paid to lipid content (0.5% to 3.6%) [5], including phospholipids (30–65%) and triglycerides, while fish oil is only comprised of triglycerides. The main phospholipid in krill oil is phosphatidylcholine, with 40% of the total fatty acids bound to phosphatidylcholine being eicosapentaenoic acid (EPA) and docosahexaenoic acid (DHA) [6]. The EPA and DHA omega-3 fatty acids found in krill oil have shown several useful pharmacological properties in the management of numerous chronic dysfunctions, including cardiovascular, neurological and inflammatory diseases, as well as the prevention of cancer and promoting gut microbiota health [7,8,9,10]. In this regard, supplementation with krill polyunsaturated fatty acids may be a natural way to relieve the symptoms of these conditions, potentially in combination with conventional therapies [11]. EPA and DHA from krill oil have also shown higher bioaccessibility than other forms of n-3 PUFAs (ethyl-ester and re-esterified omega 3), demonstrating similar benefits, but at smaller dosages [12].

Krill oil was authorized in 2008 by the U.S. Food and Drug Administration (FDA) as GRAS (Generally Recognized as Safe), was approved in Europe by EFSA as a novel food in 2009 and was also approved in China in 2014. Finally, krill oil was authorized by EFSA for pregnant and lactating women in 2014.

This review aims to provide an overview of the chemical composition, bioavailability and bioaccessibility, mechanism of actions, classic and non-conventional extraction techniques, health benefits and current applications of krill oil underlying the future perspectives of this nutraceutical.

## 2. Krill Oil Composition

Krill oil composition provides a large variety of substances beneficial to health which justifies its use as a novel food ingredient for pharmacological and nutraceutical applications in different pathological conditions, such as cardiometabolic and neurodegenerative diseases. Generally, although the content of the different classes of nutritional components is influenced by the extraction technologies, krill oil contains a high level of n-3 PUFAs and phospholipids (PLs), and minor components such as vitamins, minerals, astaxanthin and flavonoids [13,14].

### 2.1. Lipid Fraction

The analysis of the lipid component of krill oil revealed a very complex composition which is characterized by the presence of polar lipids representing the major lipid class in krill oil, followed by triacylglycerols (TAG) [15,16]. Many factors have been reported to influence the specific composition of the lipid fraction, for example interannual environmental changes, seasonal variation, krill sample variety and sexual maturity of krill samples as well as transportation process, storage conditions and pretreatment methods [17]. For example, larger amounts of FFAs have been obtained by dehydrating krill through hot air [18]. Many studies have analyzed krill oil composition through different methods of analysis [19,20]. The range of krill oil PLs ranges between 39.9% found in gravid females of *Euphausia superba* in South Georgia and 80.7% in krill oil found in Aker BioMarine (Table 1). This content varies depending on analysis methods and sample variety. Other differences in krill oil composition have been found considering feeding behavior, krill age and regions. Higher PL levels have been found in ovarian tissues and in gravid females compared to muscle tissues. Another important factor leading to variation in krill composition is extraction methods. A larger amount of PL can be obtained by using ethanol and isopropanol rather than acetone and hexane. PE and phosphatidylcholine (PC) results in the most abundant types of phospholipids ranging from 44.58–99.80%, with the low end of this range reported by Araujo and colleagues using HPTLC as the analysis method and the high end of the range described by Castro-Gomez and coworkers in 2015 using HPLC-ELSD as the analysis method. Phosphatidylethanolamine (PE), even if less abundant, has been found as 0.20% to 24.74% of total PLs [21]. Interestingly, in studies that reported a minor presence of PC, a higher amount of lysophosphatidylcholine (LPC) has been described ranging from 43.3–44.4%, probably due to PC hydrolysis caused by inappropriate storage or inadequate treatment of krill samples [22]. Other important components have been described sometimes in small amounts (less than 10% of total PLs) including phosphatidylglycerol, sphingomyelin, cardiolipin phosphatidylserine and phosphatidic acid. Due to the high content of PC, krill oil is now considered a very promising marine supply of PLs and an alternative to PLs deriving from vegetable oils, egg yolk and dairy products [23].

It is worth noting that the PL fraction obtained from the krill lipid had much higher percentages of PUFA and n-3 PUFA [24,25]. In particular, 31.13% of EPA and 14.87% of DHA were measured in the PL fraction, while only 3.17% of EPA and 1.5% of DHA were found in the TAG fraction. However, as reported by Paluchová and colleagues, the TAG lipid class containing esterified DHA proved to be the best substrate for a better bioavailability of DHA for polyunsaturated fatty acid esters of hydroxy fatty acids (FAHFA) synthesis. This is crucial for selection of novel food sources, which could stimulate endogenous synthesis of functional lipids from a nutritional point of view [26].

### 2.2. Fatty Acid Composition

As reported by the Codex Alimentarius Commission (STANDARD FOR FISH OILS CXS 32 9-2017 Adopted in 2017), the most abundant fatty acids in krill oil that have been described are C14:0 myristic acid (5.0–13.0), C16:0 palmitic acid (17.0–24.6), C16:1 (n-7) palmitoleic acid (2.5–9.0), C18:1 (n-7) vaccenic acid (4.7–8.1), C18:1 (n-9) oleic acid (6.0–14.5), C20:5 (n-3) eicosapentaenoic acid (14.3–28.0) and C22:6 (n-3) docosahexaenoic acid (7.1–15.7) (Table 2). DHA and EPA are known as n-3 PUFAs and play a fundamental role in mediating beneficial effects in different mammalian systems [29]. In general, consuming fish oil represents a daily practice for increasing EPA and DHA intake. Since EPA and DHA krill oil content are similar to other common fish oils (anchovy, tuna or salmon), consuming krill oil may represent a potential alternative for a nutritional approach as a dietary supplement [31]. Interestingly, krill lipid fraction is characterized by a higher amount of n-3 PUFA and very low levels of saturated fatty acid (SFA) and monounsaturated fatty acid (MUFA) than TAG in fish oil [32]. Indeed, EPA and DHA (respectively 31.13% and 14.87%) have been mainly found in PLs fraction, while only 3.17% of EPA and 1.5% of DHA were present in the TAG fraction [33]. This composition has been confirmed by many studies, which demonstrated that n-3 PUFAs in the PLs fraction are characterized by a higher quantity of EPA and DHA, significantly improving the bioavailability of these two pharmacological and nutraceutical components, compared to EPA and DHA contained in TAG fraction [34,35].

### 2.3. Astaxanthin

One of the most important minor components of krill oil is represented by astaxanthin, a carotenoid that has been characterized in different algae and marine animals [36]. This compound is responsible for the typical dark red color of krill oil and is endowed with potent antioxidant properties, even more than other carotenoids such as zeaxanthin, lutein, canthaxanthin, β-carotene and α-tocopherol [37]. Generally, the amount of astaxanthin in krill oil ranges from 40 to 5000 mg/kg and depends on intrinsic features of krill (for example raw material, krill species) or can vary due to extraction and analysis methods [38]. Indeed, as reported in the below section, a high concentration of astaxanthin can be achieved by using acetone as the extraction solvent [39]. Chemically, astaxanthin can be found in krill oil as a fatty acid ester. In particular, 51% of total astaxanthin is present as diester, 43% as monoesters and only 6% as free astaxanthin [40]. The main fatty acids that are conjugated with astaxanthin are myristic acid, palmitic acid, palmitoleic acid, vaccenic acid, arachidic acid, eicosapentaenoic acid and docosahexaenoic acid [41]. Furthermore, three different isomers of astaxanthin have been identified by Grynbaum and colleagues, such as (all-trans) astaxanthin, which represents the most abundant isomers, (13-cis) astaxanthin, and (9-cis) astaxanthin [41].

### 2.4. Sterols

Krill oil also contains an important fraction of sterols that range between 2.3–3.9% of total lipids [42]. Desmosterol and cholesterol represent the most abundant sterols of the total sterols [30]. Desmosterol is the precursor of cholesterol and represents 1.70–18.63% of total sterols [17]. The component variation is due to the different food intake of krill since crustaceans are not able to endogenously synthetize sterols but are supplied by diet and phytosterol dealkylation. Cholesterol has been reported as 81.33–82.34% of total sterols with a concentration of 18.95 to 31.96 mg/g of oil [43]. Compared to other fish oil, cholesterol amount in krill oil is even higher than hoki oil, which contains 5.15 mg/g of oil, 2.04 mg/g of tuna oil and 11.81 mg/g of egg yolk oil [17]. Considering the potential nutraceutical application of krill oil, a higher content of cholesterol may represent a matter of concern due to the onset of cardiovascular diseases, for example atherosclerosis, in a cholesterol rich diet. However, extraction methods could limit the content of total cholesterol in krill oil as reported by Bruheim and Cameron who demonstrated that using pure ethanol instead of ethanol mixed with water significantly reduced the cholesterol concentration. Minor percentages are represented by other sterols found in krill samples in 1977, such as 24-nordehydrocholesterol (0.1–1.7%), trans-dehydrocholesterol (1.1–1.5%), brassicasterol (0.5–1.7%), 24-methylenecholesterol (0.1–0.4%), and two stanols (0.1–0.2%) [44].

### 2.5. Vitamins

Krill oil contains a high amount of α-tocopherol (Vitamin E) which is characterized by a potent antioxidant effect. The concentration of this isoform ranges between 14.74 to 63.0 mg/100 g of oil and represents 90% of total tocopherols, while the other homologues of tocopherols (β-, γ-, δ-) are present only in traces (γ (0.25 to 3.67 mg/100 g of oil) and δ-tocopherol (0 to 0.65 mg/100 g of oil)) [45]. Besides vitamin E, vitamin A has also been found in frozen krill (about 0.11 mg/100 g of wet weight) [21]. The presence of vitamin A in krill samples represents an important feature contributing to making krill oil a very promising food supplement for the regulation of human immune function and for counteracting some infectious diseases. Depending on extraction methods, the vitamin A content of krill oil ranges between 16.4 to 28.5 mg per 100 g of krill oil, a concentration which is significantly higher than tuna oil (11.1 mg/100 g of oil) [45].

### 2.6. Flavonoids

In general, flavonoids represent a large variety of compounds with a similar chemical structure and are endowed with antioxidant activities, antibacterial and immunomodulatory effects. They also exert cardio protection and reduce the production of proinflammatory mediators. Krill oil has been reported to contain a particular flavonoid whose chemical structure is similar to 6,8-di-C-glucosyl luteolin. As reported by Sampalis and colleagues, by using specific extraction methods, a krill extract containing a significative level of flavonoid (7 mg/100 mL) can be obtained and used for protecting the skin from ultraviolet B (UVB) radiation [46]. Although some studies report that the C-glycosylation of flavonoids in krill oil could improve their antioxidant and anti-diabetic effects, there are no direct data on the use of krill oil for this purpose.

### 2.7. Minerals

Other than the organic compounds responsible for the pharmacological activity of krill oil, there is also a large quantity of minerals that have been characterized in some krill samples. Indeed, one of the most abundant minerals contained in krill is calcium (1322 mg/100 g) which can be exploited for bone health, phosphorus (1140 mg/100 g), and magnesium (360 mg/100 g) [30]. Other minerals are contained in minor quantities such as zinc, selenium and potassium. Besides these essential elements, krill is characterized by a large quantity of fluoride (2400 mg/kg dry matter) which may cause skeletal fluorosis if its intake is high. However, the major quantity of fluoride is contained in the krill exoskeleton which could be removed during krill oil extraction to avoid excessive fluoride content reaching low fluoride levels (<0.5 ppm) [47].

## 3. Mechanism of Action

Due to the complex composition of krill oil, which contains structurally different chemical compounds such as PUFAs, flavonoids, astaxanthin and vitamins, the pharmacological effects that have been described are ascribable to multiple mechanisms of action. Krill oil is characterized by a high quantity of (n-3) PUFA (mainly EPA and DHA) which are natural PPAR’s ligands responsible for the activation of PPAR [33]. These transcription factors play a fundamental role in regulating cell and tissue behavior to different stimuli. Generally, PPAR forms a heterodimer with the retinoic-X-receptor whose ligand is represented by cis-9-retinoic acid [48]. PPARα and PPARγ are the most investigated isoforms of PPAR. PPARα is mainly expressed in hepatic cells and regulates lipid accumulation [49], while PPARγ has been mainly described in adipose tissues where it promotes insulin sensitivity, adipocyte differentiation and regulates metabolic responses, fat storage and energy homeostasis [50]. Furthermore, PPARγ has also been described in inflammatory cells, where it controls the release of proinflammatory mediators and promotes anti-inflammatory effects [51]. The PPARγ activation occurs in the cells and the uptake of EPA and DHA seems to be due to the expression of FAT/CD36 (a transmembrane fatty acid transporter) [52]. Intriguingly, PPARγ also regulates the expression of FAT/CD36 itself, indicating that n3-PUFA can increase their own uptake inside the adipocytes, promoting the production of adiponectin [53]. The involvement of PPARγ have been demonstrated using PPARγ antagonists (e.g., bisphenol-A-diglycidyl ether or GW9662) that suppressed the secretion of adiponectin [54]. Furthermore, n-3 PUFA are able to activate PPAR through a non-covalent interaction, promoting the reduction of inflammatory responses with a consequent reduction of the release of TNFα and IL-6 after LPS stimulation [55].

Another important target mediating the pharmacological effects of krill oil is represented by G protein-coupled transmembrane receptors (GPCRs) which are involved in the regulation of many metabolic processes. In particular, EPA and DHA activate GPR120 (also known as FFA receptor 4; FFAR4) leading to the increase of intracellular cAMP level and Ca2+ concentrations, consequently promoting the phosphorylation of extracellular signal-regulated kinases 1/2 (ERK1/2) [56]. Since EPA and DHA are involved in the regulation of inflammatory processes mainly in adipose tissue, the involvement of GPR120 has been investigated. In particular, DHA inhibited the IKK (Inhibitor of κB kinase) complex activation and JNK phosphorylation resulting in a reduction of TNF-α release in macrophages treated with LPS [57]. The involvement of GPR120 has been confirmed by GPR120 knockdown. In addition, DHA has been reported to facilitate the formation of GPR120 and β-arrestin2 complex (GPR120-βarr2) that blocks the pro-inflammatory stimulus due to LPS exposure [58]. The GPR120-mediated anti-inflammatory effects of DHA and EPA have also been confirmed in 3T3-L1 adipocytes, resulting in a significant reduction in MCP-1, IL-1β and TNF-α gene expression [58].

The inflammatory process is mainly regulated by NF-κB. Once this transcription factor is activated after IκB phosphorylation due to external stimuli such as UV radiation, endotoxins, oxidative stress, saturated fatty acids, it is able to translocate into the nucleus. It can then promote the production of several pro-inflammatory mediators, adhesion molecules, COX-2, and inducible NO synthase [59]. EPA and DHA, as reported above, reduce the production of a variety of pro inflammatory molecules, such as TNFα, IL-1, IL-6, IL-8, and IL-12 and limit the transcription of those enzymes involved in the inflammatory process including inducible NO synthase and COX-2 in different cell lines (for example endothelial cells, macrophages and monocytes) [60]. This effect seems to involve the reduction of IκB phosphorylation and consequently the reduction of the activation of NF-κB in a GPR120 and PPARγ dependent manner. Indeed, PPAR physically interacts with NF-κB, avoiding its translocation into the nucleus. Furthermore, NF-κB activity is also related to GPR120 since DHA strongly inhibited IKK activity via GPR120 in both stimulated macrophages and adipocytes [61].

In addition, treatment with n3-PUFA limited NF-κB DNA binding activity in adipocytes, macrophages and THP-1 monocytes stimulated with LPS, which prevents the production of IL-6, IL-1β and TNF-α [62]. Intriguingly, as along with NF-κB DNA binding activity being reduced, the authors demonstrated that PPARγ DNA binding activity was also significantly abolished, providing evidence of the tight connection between PPARγ and NF-κB activity in regulating the inflammatory processes. Alongside the reduction of pro inflammatory mediators, treatment with EPA and DHA promoted the release of IL10 in 3T3-L1 adipocytes [63]. IL10 is an important interleukin that is involved in the anti-inflammatory response and inhibits IKK, preventing NF-κB DNA binding activity and PPARγ binding motif. This demonstrates that n3-PUFA may regulate NF-κB activity by inducing IL-10 expression in a PPARγ dependent manner [64].

Besides the high content of EPA and DHA, krill oil also contains potent antioxidant molecules. In particular, many studies have demonstrated that the presence of astaxanthin is responsible for the potent antioxidant effect and potentially the well-known anti-inflammatory properties of EPA and DHA. Oxidative stress is the leading cause of many pathological conditions by triggering the activation of important proinflammatory intracellular pathways which feed a vicious circle. This is especially true in neurodegenerative processes and in cardiovascular diseases characterized by endothelial dysfunction. Preventing the excessive production of reactive oxygen species (ROS) through a nutraceutical approach may be a promising strategy to manage several pathological conditions. In this context, Nrf-2 is one of the main transcriptional factors controlling antioxidant machinery. Indeed, its activation is reported to exert beneficial effects through the increased production of direct antioxidant molecules as well as the hyper activation of antioxidant enzymes SOD, CAT, and GPX [65]. Nrf-2 is one of the main targets of the antioxidant effect of astaxanthin, which induces Nrf2–ARE-mediated antioxidant enzymes in different in vitro models. Astaxanthin reduced oxidative stress in neuronal cells exposed to doxorubicin results in an increase in cell viability and reduction of pro inflammatory mediators [66]. Similarly, astaxanthin protected human mesangial cells exposed to high glucose exert an anti-inflammatory and anti-oxidant effect [67].

Oxidative stress, beyond promoting the inflammatory response, is also responsible for insulin resistance due to the activation of several kinases, among which JNK promotes the phosphorylation of IRS-1, inhibiting its activity and preventing its interaction with the insulin receptor. In addition, elevated levels of ROS cause the degradation of the GLUT4 vesicle, dramatically decreasing glucose uptake [68]. The potent antioxidant effect of astaxanthin facilitates insulin secretion, accelerates glucose metabolism and improves insulin sensitivity, IRS-1 activation, Akt phosphorylation, and GLUT4 translocation in skeletal muscle. This leads to increased insulin sensitivity and a decrease in blood glucose level, which in turn paves the way for using the astaxanthin nutraceutical supply for the management of type 2 diabetes [69] (Table 3).

## 4. Krill Oil Extraction Technologies

**Figure 1:**
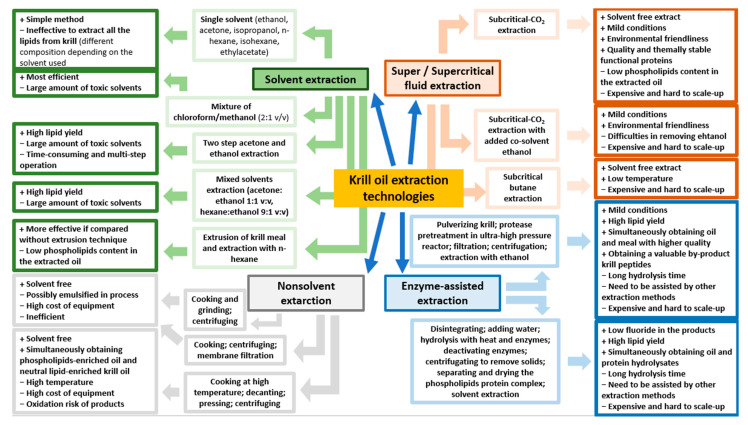
The advantages and disadvantages of krill oil extraction technologies.

Krill oil can be extracted from different biomasses of fresh krill and dried krill [70,71]. However, fresh krill contains high levels of proteolytic enzymes that induce the rapid autolysis of the crustacean. For this reason, it is necessary to process krill immediately after it is caught [72]. The extraction techniques (Figure 1) can be divided into classic and non-conventional methods. Conventional techniques include several solvent extractions, while the innovative methods include non-solvent extractions, super- and sub-critical fluid extractions and enzyme-assisted extractions.

### 4.1. Conventional Extraction Techniques

Solvent extraction is the most conventional method to obtain krill oil. However, different solvents must be used to extract all the lipids from krill because of the diversity of the polarities of the lipid compounds in the biomass. As reported in a study by Xie et al., ethanol and isopropanol can extract most of the phospholipids, although the obtained krill oil is lacking in other lipid components [21]. Hexane is the most common solvent for vegetable oil production due to its low cost, high extraction efficiency and because it is easily eliminated and leaves few traces of residues in food sources. Nevertheless, data on the use of hexane for krill oil production has demonstrated that this solvent only has moderate PL-extracting ability [28,73].

Acetone has proven to be effective for the extraction of minor molecules from krill, but not for PLs. In this regard, a combination of nonpolar and polar solvents is a good compromise for the extraction of both the PLs and other molecules from krill [74]. Two-step acetone and ethanol extraction techniques are the most common methods used for krill [71], as other methods, including the Folch method (which is popular for lipid extraction from animal tissues), are not commercially feasible because of the toxicity of the solvents used (e.g., chloroform and methanol) [75]. Similar results have been obtained using a single-step extraction method with a mixture of acetone and ethanol as the solvents (1:1, v/v) [76]. Moreover, lipid efficiency can be improved by combining the extrusion pre-treatment of krill meal with the solvent extraction technique [77].

Environmental concerns, which have a real impact even in krill oil production, are one of the main limitations with the use of solvents. In addition, this technique is time-consuming and labor intensive because of the multiple extraction and evaporation steps. However, it remains the most economical and easily scalable.

Krill oil can also be extracted using non-solvent techniques including mechanical pressing, which is commonly used for oilseed extraction, such as in sesame oil and sunflower oil [78]. However, this method is inefficient compared to solvent extraction, for two reasons in particular: the low lipid content present in fresh krill (0.5–3.6%) and the difficulty of the mechanical processing used for krill [77].

**Figure 2:**
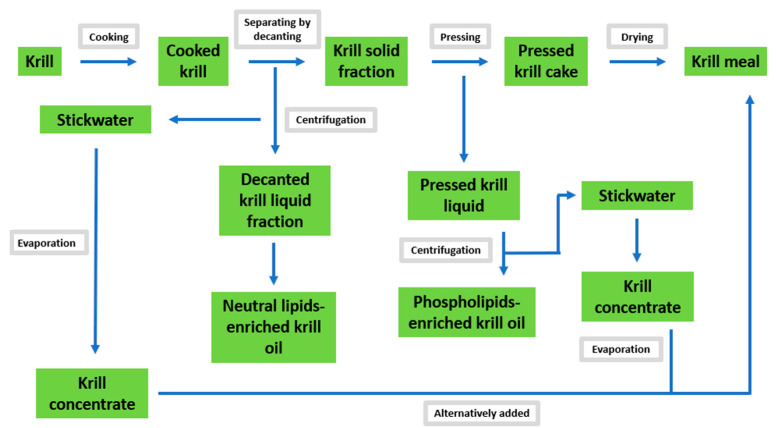
An overview of non-solvent extraction technique (Adapted from Katevas et al. [70].

Non-solvent sequential procedures, such as cooking, decanting, pressing and centrifuging, are able to successfully separate krill oil from the mixture (Figure 2). In fact, the release of PLs from the lipid fraction of fresh or defrosted krill—after conventional grinding into a slurry which involves mechanical disruption procedures and centrifugation—does not facilitate the complete separation of the oil fraction due to the amphipathic nature of PLs, which act as emulsifying agents in the formed slurry [79]. Katevas et al., have developed a method that can extract both the PL fraction and the neutral lipid-enriched krill oil. In this method, the first cooking step is conducted at 90° C without grinding and agitation, thus avoiding the emulsification process. The same authors concluded that fresh krill is a better option than defrosted krill as it was able to avoid or reduce the emulsification process, which may occur in defrosted krill due to the formation of ice crystals (after freezing) and the consequent disruption of krill tissue [70].

Although the non-solvent extraction technique avoids the use of solvents, making it safer and more eco-friendly than solvent-extraction methods, it presents many drawbacks. First, it is poorly scalable because of the high investment in equipment and the high costs of energy required. Secondly, the cooking procedure at high temperatures may induce lipid oxidation in the products. Last but not least, this technique has only been observed to give a total lipid yield of 2.2% and does not extract all of the available oil from krill. For these reasons, mechanical separation can be used to initially obtain part of the krill oil, but also to produce krill meal, which can be subsequently treated with other techniques including supercritical fluid extraction and solvent extraction [80].

### 4.2. Unconventional Extraction Techniques

The supercritical fluid extraction method is an unconventional extraction technique that can be used for krill oil extraction and is known to be solvent-free [81]. It is considered environmentally friendly as it uses supercritical carbon dioxide, which is safe, non-toxic and chemically inert. Nevertheless, carbon dioxide is not an ideal molecule for the extraction of all the lipid content from krill as it fails to extract PLs [82]. For this reason, the addition of 5–20% of ethanol to supercritical carbon dioxide extraction has been tested and demonstrated to improve PL solubility and thus lipid recovery [83]. The use of this technique presents some issues, including its limited processing capacity, the high cost of the equipment and the risk of de-solvation by the liquid state of ethanol at 25 °C.

Subcritical fluid extraction with compressed butane and propane, performed at low temperature and pressure, is commonly used for oil extraction [84]. In a study conducted by Xie et al., the use of subcritical butane (30 °C, 0.3 to 0.8 MPa) gave results that are comparable to those of hexane in terms of yield and krill oil quality, although it was faster and the extraction process required less solvent [73]. In addition, in a study by Sun et al., subcritical butane extraction was proven to give the extract that was richest in astaxanthin and tocopherol, and the lowest in oxidation degree, compared to the solvent extraction technique [85]. However, even in this case, the high cost of equipment and limited processing capacity are the main issues for a successful scale-up process.

Enzyme-assisted extraction is a novel pre-treatment technique than can increase lipid yield, as it can release the bound molecules using specific enzymes (e.g., amylase, protease, glucanase, cellulase and pectinase) [86]. These enzymes can selectively enhance the extractability of lipid compounds, improving the breakdown of cell walls and destroying lipid bodies. The high quality of the extracted oil and the mild process conditions also make this technique attractive for the extraction of krill oil [75]. Bruheim et al., have obtained satisfactory results with enzyme-assisted extraction for krill oil. After an initial krill-disintegration process, the small particles were treated with water and heat and the hydrolytic enzymes were then added to hydrolyze the material and improve lipid extraction. Finally, the enzymes were deactivated and, after removal of the solid material, the PL-protein complex was separated and dried, and the krill oil was extracted [75]. Finally, in a study by Lee et al., a combination of an enzyme-assisted extraction process and an ultrahigh-pressure (10 to 300 MPa) reactor made krill easily liquefiable and ensured full contact with the proteases [87].

The high costs and longer hydrolysis times are still challenging for the use of this technique in large-scale industrial applications.

## 5. Bioavailability and Bioaccessibility of Krill Oil Omega-3

Several studies have investigated the effects of krill oil supplementation on health, and these effects appear to be superior to those of fish oil [88,89]. The superiority of krill oil has been attributed to the higher bioavailability of EPA and DHA, which are in the form of PL [90]. Nevertheless, most of the RCTs conducted to date did not use the same doses of EPA and DHA (for the same outcomes) from krill and fish oil and ignored the differences between the bioactive substances in fish and krill oil [91].

In fact, the minor components contained in krill oil, such as astaxanthin, alpha-tocopherol, vitamin A and flavonoids, can exert pleiotropic activities and have a positive impact on health, in addition to probably improving the bioaccessibility of EPA and DHA. In this regard, a study by Kohler et al., s reported that EPA and DHA from krill meal had lower bioavailability than krill oil, but the same as fish oil. This study underlines the potential role of minor lipophilic molecules in improving the bioaccessibility, and thus the bioavailability, of EPA and DHA from krill oil [92]. However, the mechanisms of action by which krill oil appears to be superior remain unknown and seem to be closely related to the extract in its entirety and the extraction method.

Long-term RCTs are needed to determine the differences in efficacy and performance of krill oil compared to fish oil. This information is currently incomplete and needs clarification, starting from the role of minor components in the extracts. In addition, EPA and DHA supplemented from fish and krill oils in comparative studies should be at the same dosages and ratio. In fact, in a study by Ramprasath et al., which reported the superiority of krill oil over fish oil (expressed as concentration of n-3 PUFA plasmatic levels), supplementation with EPA and DHA was 777 mg for the krill oil group and 664 mg for the fish oil group [91]. Ulven et al., have demonstrated a change in the EPA/DHA ratio after supplementation with 543 mg of krill oil and 864 mg of fish oil (EPA/DHA ratio: 1.74 for krill group and 1.12 for fish oil group) [12]. Weather this improvement of EPA/DHA ration is an advantage in terms of health effect it is yet to be fully demonstrated.

## 6. Krill and Metabolic Disorders

Dietary supplementation with omega-3 fatty acids has been demonstrated to be beneficial for the prevention and/or treatment of cardiovascular diseases (CVD) [93] and possibly other inflammatory and neurological disorders [94,95]. Moreover, increased consumption of EPA and DHA may also be of clinical significance in the prevention and reversal of insulin resistance [96].

The American Heart Association (AHA) recommends a daily intake of at least 1000 mg of omega-3 fatty acids to minimize risk factors associated with CVD, even for patients at high risk of developing CVD. Therefore, despite the great abundance of omega-3 fatty acids available in fish oil, the delivery of fatty acids and presence of astaxanthin in krill oil may provide superior health benefits and meet the AHA recommendations [97].

Krill oil is endowed with a unique chemical composition. Unlike fish oil, it is rich in omega-3 fatty acids present in the form of phospholipids rather than triglycerides. This may be biologically and therapeutically significant, since phospholipids are well-absorbed by the intestine and they are readily incorporated into cell membranes, suggesting that they could be endowed with a more favorable pharmacokinetic profile [98]. Moreover, supplementation with krill oil gives the advantage of not only supplying n-3 PUFAs, but also choline, which is an essential nutrient, since that it is needed in the synthesis of neurotransmitters (acetylcholine) and phospholipids and is important in the transport of lipids and reduction of homocysteine [23]. In addition, krill contains several endogenous antioxidants including astaxanthin (which is responsible for the deep red color), the preservation of krill oil against oxidation and it has potential health-promoting properties [99].

Maki and colleagues observed that a supplementation for four weeks with Antarctic krill oil (2 g/day) increased plasma concentration of EPA and DHA [100]. This suggests it could be used similar to the way fish and fish oil are used, as it is also rich in long-chain omega-3 polyunsaturated fatty acids which contains cardiovascular risk. In this context, supplementation with krill can also represent a promising approach to ameliorate obesity and obesity-associated diseases. To date, preclinical evidence has been collected on the positive impact of krill oil in conditions of metabolic disorders.

It has been also demonstrated that plasma EPA level was significantly greater following krill oil treatment when compared to fish oil treatment. Significant remodeling of the plasma lipidome was observed after four weeks of treatment with krill oil (containing 1.27 g/day of long-chain omega-3 polyunsaturated fatty acids) if compared to fish oil (containing 1.44 g/day of long-chain omega-3 polyunsaturated fatty acids), with a clear differentiation in their effects on different plasma lipids species. In particular the authors reported that more than 38% of the lipids species increased following krill treatment, while only 12% increased when fish oil was used as supplementation [101]. Similar results have been obtained in healthy women over a five hour postprandial period. Clear differences between krill oil and fish oil supplementations in the postprandial period were reported. The most noticeable changes were revealed in diacyl-phospholipids and ether-phospholipids [102].

Finally, subchronic toxicity and genotoxicity studies in rats confirm that krill oil is well-tolerated and seems to be safe with a daily supplementation of 5% [103].

In male Sprague Dawley rats who were fed a high-fat diet (HFD) for two weeks, the consumption of krill oil significantly reduced serum lipids, and the two highest krill oil doses (100 and 200 g/L) also significantly increased HDL levels [104].

In 2009 Cohn’s group demonstrated that supplementation of krill oil to HFD mice for eight weeks, dose-dependently reduced hepatic triglycerides, cholesterol and serum, as well as total cholesterol and glucose. Unfortunately, this study was unconclusive regarding the putative mechanism. The peroxisome proliferator-activated receptor (PPAR)α which is supposed to be critical to promote β-oxidation at the liver level, did not significantly change which means it was not involved [105].

The intake of a powder isolated from Antarctic krill showed to ameliorate the hepatic metabolism in a transgenic mouse model of chronic inflammation, for expressing the human tumor necrosis factor-alpha (hTNFα) gene. Lower hepatic and plasma triacylglycerol levels, as well as hepatic gene expression of sterol regulatory element binding transcription factor 2 (SREBP2) and enzymes involved in cholesterol synthesis were found. In addition, genes involved in lipogenesis and glycerolipid synthesis were down-regulated and β-oxidation was promoted, confirming the capability of this product to increase the hepatic lipid catabolism and suppress lipidogenesis. Finally, krill powder reduced endogenous TNFα in the liver, indicating anti-inflammatory effects [106].

In 2020, Saito et al. evaluated the effects of 8-HEPE-concentrated material from Pacific krill on dyslipidemia and hepatic steatosis in low-density lipoprotein (LDL) receptor-deficient (LDLR-KO) mice. Very interestingly, they observed that over 18 weeks a supplementation of a typical western diet with Pacific krill (8-HEPE (100 mg/kg), but not EPA or DHA) improved the lipidic profile (reducing LDL and total cholesterol levels and increasing HDL levels) and reduced hepatic triglyceride levels [107]. This led to the hypothesis that eicosapentaenoic acid (EPA) and 8-hydroxyeicosapentaenoic acid (8 HEPE) have more positive effects on the metabolic syndrome by activating the peroxisome proliferator activated receptor (PPAR α) in the liver.

In addition, in dyslipidemic and diabetic non-human primates, the daily intake omega-3 phospholipids purified from krill showed a positive impact on CVD risk factors by reducing total cholesterol, LDL-cholesterol and triglycerides and increasing HDL-cholesterol, if used at the dose equal to 150 mg/Kg/day [108].

Runbland and colleagues reported a randomized control study carried out on 36 individuals who were divided in three groups: fish group, krill group and control group. For eight weeks, krill and control groups received capsules containing oil, while the fish group was invited to consume lean and fatty fishes based on dietary guidelines. As expected, the levels of EPA and DHA increased in the fish group and krill group, whereas docosapentaenoic acid (DPA) increased only in the krill group. However, the overall differences between the three intervention groups were significant for EPA (*p* < 0.0001), DPA (*p* < 0.001) and DHA (*p* < 0.001). In general, the authors observed a great variability among the participants, however a tendency towards a decrease in total lipids and triacylglycerols was observed in the fish and krill groups with the largest VLDL levels. In agreement with previous works in animal models, in which a down-regulation of the genes involved in gluconeogenesis has been demonstrated [109,110], as well as in clinical studies [111], they reported a significant reduction in fasting blood glucose in the krill group compared with the control group. This result is strongly predictive of a reduction of CVD risk [112].

In humans with borderline or high triglyceride levels (range between 150–499 mg/dL), the treatment with krill oil at the dose of 0.5, 1, 2, or 4 g/day for 6 and 12 weeks could be efficient to reduce triglycerides. Nevertheless, the great heterogeneity of the selected sample impeded to have a clear view of the real nutritional value [113].

Cicero et al., published a randomized cross-over clinical trial in which 25 moderately hypertriglyceridemic subjects (150–500 mg/dL) were treated with omega 3 ethyl ester (2000 mg/day) or krill oil (1000 mg/day) for four weeks. Only the krill oil treatment significantly improved HDL and apolipoprotein AI levels, compared to the values measured both at baseline (*p* < 0.05) and at end of treatment in the group supplemented with esterified omega 3 (*p* < 0.05). Both treatments were able to significantly reduce high-sensitivity C-reactive protein (hs-CRP) levels from the baseline (*p* < 0.05), but krill oil improved it more efficaciously than esterified omega 3 ethyl ester group (*p* < 0.05) [114].

A reduction in body weight because of krill oil supplementation in obesity models has been reported in some animal studies. Sun and colleagues observed that an Antarctic krill oil, extracted from dry krill using an innovative procedure of hot pump dehydration combined with freezing-drying, was endowed with anti-obesity effects in metabolic disorder conditions. In particular, supplementation for 12 weeks improved dyslipidemia, fatty liver and glucose metabolism in C57BL/6J mice fed with HFD. Krill oil also reduced body weight gain, reduced fat accumulation in adipose and liver tissue, lowered serum density of lipoprotein-cholesterol (LDL-C) content and ameliorated glucose tolerance. In addition, krill oil feeding also reduced oxidative damage in the liver [115].

In another study, rats were fed a control diet, an HFD or an HFD supplemented with 2.5% krill oil for 12 weeks. Krill oil significantly prevented increased body weight in the HFD group [105].

An improvement of insulin sensitivity and secretion after administration of krill oil (600 mg/day) has also been seen in an obesity model of castrated male New Zealand white rabbits [110]. Expression levels of key enzymes involved in the β-oxidation and lipogenesis were different after krill oil feeding for 8 weeks, compared to placebo, which ultimately led to decreased fasting blood glucose and improved glucose tolerance in the rabbits.

Further evidence suggested that omega-3 phospholipids of krill oil enhanced intestinal fatty acid oxidation and could contribute to the anti-steatotic effects in obese mice, meaning there could exist an axis microbiota-intestine-liver [116]. Indeed, omega-3 alleviated hepatic steatosis in various rodent models of obesity even in exacerbated hepatic steatosis conditions, in which an HFD was combined with thermoneutral animal housing (i.e., ambient temperature approximately 30 °C) [117].

Several authors highlighted that omega-3 supplemented by krill oil is even more effective, mainly phosphatidylcholine -rich phospholipids when compared to the same dose of the triacylglycerol form. It has been observed that omega-3 phospholipids contribute more effectively to improve glucose intolerance and insulin resistance in dietary obese mice when compared to their triacylglycerol form [116]. The reason for this is likely due to the amelioration of bioavailability; EPA and DHA present in fish and fish oil are almost exclusively in triacylglycerol form, while in krill oil up to 65% of EPA and DHA occur in phospholipids [118].

Krill oil was also found to directly influence cardiac remodeling and function in an experimental myocardial infarction (MI). In such experimental conditions, rats were randomized in krill oil or control groups for 14 days before the induction of MI. Seven weeks after the MI induction, the echocardiography showed a significant attenuation of left ventricular (LV) dilation in the group pre-treated with krill oil. Attenuated heart and lung hypertrophy and reduced mRNA levels encoded classical markers of LV stress, including matrix remodeling and inflammation [119].

Krill oil has been also associated with moderate improvement in endothelial dysfunction and HDL, two known CVD risk factors, in patients with type 2 diabetes. In 34 participants with type 2 diabetes, an improvement of their endothelial function and a reduction in blood C peptide levels and HOMA scores were reported after four weeks of supplementation with krill oil (1 g/day in PUFA) when compared to the olive oil group. There were differences in weight loss between krill oil and olive oil after 17 weeks, though if compared with their respective baseline measurements, the participants of each group had a statistically significant improvement in endothelium [120].

A close relationship between krill oil consumption and reduction of circulating levels of endocannabinoids 2-arachidonoylglycerol (2-AG) and N-arachidonoyl-ethanolamine (AEA) has been highlighted [116].

In obese subjects, the endocannabinoids are elevated in the blood and this phenomenon appears to be due to changes in expression of adipose tissue metabolizing enzymes. Moreover, endocannabinoids are made by enzymatic reactions from arachidonic acid; hence, the more omega-6 arachidonic acid (ARA) available, the more endocannabinoids can be made. On the other hand, an increased intake of omega-3 might help to counterbalance a disturbed omega-3 to omega-6 ratio and result in lower endocannabinoid levels that may positively affect membrane signaling and energy metabolism. Recently, Di Marzo and Silvestri brought to light the existence of a triangle among the lifestyle–gut microbiome–endocannabinoid system and its crucial role in the development of metabolic syndrome [121].

Interestingly, krill oil supplementation (dose ranging 1.25–5%) led to a significant decrease in AEA, as compared to controls [122]. According to previous preclinical evidence, Berge and colleagues demonstrated that besides a reduction of plasma triglycerides, supplementation with krill powder (4 g/day per os) contributed to a reduction in 11 obese men’s anthropometric parameters and blood endocannabinoid (AEA and 2-AG), whose levels were correlated with high levels of triglycerides and were responsible for hyperactivity of the cannabinoid system which feeds metabolic dysfunction. Indeed, the endocannabinoid system is deeply involved in the regulation of the homeostasis of body composition by regulating food intake and energy expenditure; therefore, this could be another mechanism through which krill may have beneficial effects on metabolism [123]. According to a previous paper, a significant reduction of the 2-AG levels has been highlighted with 2 g/day of krill oil (providing 309 mg/day of EPA/DHA 2:1), although no significant effect on anthropometric parameters has been observed [124].

## 7. Krill and Inflammatory Bowel Diseases and Gut Microbiota

It is well known that obesity and its complications, such as insulin resistance, hyperlipidemia and atherosclerosis caused by HFD are often accompanied with alteration in gut microbiota, in particular an increase of pro-inflammatory/pathogenic bacteria. In 2017, Cui et al. observed that treatment with fish oil (600 μg/g/day), krill oil (600 μg/g/day) and their mixture (300 + 300 μg/g/day) for 12 weeks led to obesity alleviation, as well as gut microbiota modulation. In fact, they reported a decrease of body weight gain, adiposity index and liver index. They also reported an increase in the abundance of some positive phyla in the gut, including *Bacteroides* and *Lactobacilli* [125]. Likewise, Lu and colleagues demonstrated that supplementation with krill oil shifted the gut microbiota composition and that it was associated with the alleviation of hyperlipidemia. According to an experimental model, mice fed for 12 weeks with a high fat and high sugar diet showed obesity and hyperlipidemia. Treatment with a high dose (600 µg/g/day) of krill oil, but not with lower doses (100 µg/g/day or 200 µg/g/day) improved the microbiotic alteration and cardiometabolic parameters [126].

Krill oil treatments for seven weeks at different dosages (100, 200 and 600 mg/kg) decreased the abundance of tyrosine consumers and increased the abundance of Lactobacillus spp. and short-chain fatty acids producers [127].

Moreover, other research points out beneficial effects of krill supplementation against inflammatory bowel diseases (IBD), including ulcerative colitis and Crohn’s disease, which share common symptoms such as bleeding, diarrhea and weight loss. In these cases, the integrity of the intestinal barrier layer and the gut microbiota play a critical role. In this regard, a mixture composed of krill oil plus probiotic *Lactobacillus reuteri* plus vitamin D has demonstrated to significantly improve clinical and histological scores, restore epithelial restitution and reduce proinflammatory cytokines in an experimental model of colitis induced by dextran sulphate sodium (DSS) treatment [128]. Of note, krill oil also appears to attenuate inflammation in an experimental model of ulcerative colitis in rats. In male rats submitted to treatment with dextran sulphate sodium (DSS), the supplementation with krill oil (5%) for 30 days preserved the colon length, which was significantly shortened in the DSS-treated compared to control animals, in line with oedema and inflammation in the colonial mucosa. Moreover, typical factors such as disease activity index (DAI) and TNF-α and IL-1β levels were positively affected by krill oil compared to DSS administration alone [129].

Suppression of the pro-inflammatory cytokines TNF and IL6 and the systemic levels of endotoxin, a marker of IBD, were also found. Recently innovative krill oil-entrapped liposomes were developed and their efficacy in an IBD model was demonstrated. The authors observed that liposomes were incorporated into the impaired enterocyte membrane, which contributed to re-establish the hydrophobic protective barrier in the inflamed/impaired region and decrease the permeation typical of IBD [130].

Liu and colleagues suggested that krill oil could contribute both to attenuating the inflammatory pathway and modulating gut microbiota through the reduction of Rickettsiales and several species of *Lactobacillus* [131].

## 8. Inflammation

### Arthritic Diseases

Based on pre-clinical studies carried out on mice experimental models of inflammatory arthritis, krill oil was supposed to have a positive effect in reducing joint inflammation more so than fish oil. In a rodent model of rheumatoid arthritis, mice fed with a krill oil diet, in which EPA and DHA were 0.44 g/100 g of krill oil diet, exhibited a decreased infiltration of inflammatory cells at the joint level, and decreased hyperplasia at the synovial layer, compared to controls [132]. Moreover, in mice transgenic for human TNF-α, while the fish oil and krill oil had a similar effect on cholesterol levels, only krill oil reduced markers of fatty acid oxidation [133]. This is consistent with the observation that fish oil and krill oil are both rich in EPA and DHA, but only krill oil naturally contains antioxidants agents like astaxanthin [134].

In a previous randomized, double blind, placebo controlled clinical trials carried out on 90 patients affected by cardiovascular diseases and/or osteoarthritis and/or rheumatoid arthritis, the effect of krill oil on C-reactive protein (CRP) and on arthritic symptoms were evaluated. Patients received krill oil 300 mg/day or placebo for 30 days and CRP and osteoarthritis symptoms were recorded at baseline and at days 7, 14 and 30. Despite the short treatment, krill oil significantly reduced CRP levels even after 7 days (about 20% of CRP reduction versus an increase of about 16% in the placebo group), reaching a higher reduction (about 30% versus an increase of CRP levels of about 25% in the placebo arm) after 30 days. Moreover, krill oil significantly reduced symptoms such as pain by about 29%, stiffness by about 20% and functional impairment by about 23%, suggesting that a 300 mg daily dose of krill oil could represent a good strategy to counteract arthritic symptoms and inflammation period [135].

In a more recent randomized, double-blind, parallel-group, placebo-controlled study, 50 adult patients affected by mild knee pain received 2 g/day of krill oil or placebo for 30 days. After 30 days of treatment, patients treated with krill oil exhibited a significant reduction of knee pain both in sleeping and in standing and the range of motion in both knees was improved [136].

These promising pre-clinical and clinical studies led Laslett and co-workers to design and start a clinical trial named “KARAOKE” (Krill oil for OA of the knee), focused on the use of krill oil to improve knee osteoarthritis (OA). This study is currently ongoing and 260 Australian patients affected by knee OA characterized by pain, synovitis and effusion will be recruited and randomized to receive 2 g/day of krill oil or placebo every day for six months. Symptoms, functionality and knee structural abnormalities will be monitored at the beginning of the study and after 6 months using validated clinical methods and magnetic resonance imaging. In particular, the primary outcomes of this study are change in knee pain and in size of knee synovitis/effusion over 24 weeks. The secondary outcomes are improvement in knee pain over 4, 8, 12, 16 and 20 weeks [11].

## 9. Neuroprotection

### 9.1. Neurodegeneration and Alzheimer Disease

The astaxanthin content present in krill oil was evaluated as a peculiar feature which gives krill oil more antioxidant properties than fish oil. In particular, the first results demonstrated that astaxanthin was able to protect the human neuronal SH-SY5Y cell line from an oxidative stimulus via its ability to act as a mitochondria protective agent [137]. In the same neuronal cell line, astaxanthin induction increased expression of the antioxidant enzyme heme oxygenase-1 (HO-1) by activation of the ERK1/2 signal pathways. This mechanism could account for the neuroprotection induced by astaxanthin against the cellular apoptosis induced by beta-amyloid (Aβ25-35). This protection was abolished by the administration of a specific ERK inhibitor [138]. Finally, astaxanthin (250–1000 nM) in primary culture of cortical neurons prevents the H_2_O_2_-induced (50 µM) reduction of cell-viability, restores mitochondrial potential and inhibits apoptosis. In a rat model of focal cerebral ischemia, in vivo, astaxanthin which was administered intragastrically at 80 mg/kg twice (5 h and 1 h before the induction of cerebral ischemia), induced a significant protection against infarct volume, improving the neurological deficit [139].

Several pre-clinical studies investigated the role of krill oil on cognitive function. In particular, Lee and colleagues evaluated the potential improvement in memory and learning due to the administration of phosphatidylserine isolated from krill (PK) in aged rats through the Morris water maze experimental model. Their results demonstrated that the administration of PK 20 or 50 mg/kg/day for 7 days significantly improved the escape latency for finding the platform in the Morris water maze compared with rats which received the 50 mg/kg/day of phosphatidylserine isolated from soybean. Moreover, the treatment with PK also improved the loss of cholinergic immunoreactivity, muscarinic receptors and choline transporters typically observed in the hippocampus of aged rats, demonstrating a neuroprotective role for PK [140]. The examination of these results on different components naturally contained in krill oil, taken together with the presence of omega-3, led Barros and colleagues to hypothesize a perspective for the use of krill oil as a neuroprotective supplement [141].

After the above reported studies demonstrating the neuroprotective effect of krill oil, more specific studies on neurodegenerative diseases were carried out on recognized animal models. Among them, Choi and co-workers administered krill oil 80 mg/kg/day for one month to a mice model of Alzheimer obtained through lipopolysaccharide (LPS) injections, 250 µg/kg, seven times daily. The team found that krill oil induced a general reduction in oxidative and inflammatory markers. Krill oil administration prevented the LPS-induced expression of the inducible isoform of nitric oxide synthase (iNOS) and of cyclooxygenase-2 (COX-2), inhibited IkB degradation suppressing the NFkB pro-inflammatory signaling and induced a decrease of ROS levels and malondialdehyde levels. Moreover, krill oil also suppressed amyloid beta (1–42) peptide generation, demonstrating a multitarget mechanism on the three main aspects which support Alzheimer disease: oxidation, inflammation and amyloid beta production [142].

A recent study, carried out on a more specific animal model of neurodegenerative diseases, represented by the senescence-accelerated prone mouse strain 8 (SAMP8) mice, demonstrated that krill oil improved both cognitive function and anxiety. In particular, the administration of a diet enriched with 1% of krill oil for 12 weeks improved SAMP-8 performances tested through the Morris water maze, the open-field test and the Barnes maze test, resulting in reduction of memory deficit and learning improvement. Moreover, by examining the hippocampus it was clear that krill oil reduced β-amyloid Aβ42 accumulation. This effect was linked to a mechanism involving an increase in activity of glutathione peroxidase and superoxide dismutase and a contemporary decrease in malondialdehyde and 7,8-dihydro-8-oxoguanine levels [143]. Interesting findings about possible use of krill oil in prevention and treatment of Alzheimer disease were recorded by Kim and co-workers through the employment of a mouse model of Alzheimer obtained by injection of amyloid Aβ25-35 in mice. After the Aβ25-35 injection, mice developed cognitive impairments but the mice receiving an oral administration (gavage) of 100, 200 or 500 mg/kg/day of krill oil for 14 days showed shorter latency in the Morris water maze test, downregulation of Bax/Bcl-2 ratio in the brain and reduced levels of ROS, malondialdehyde and NO [89].

Despite the evidence obtained in pre-clinical studies, the clinical trials studying the effect of krill oil on human brain are very poor. In particular, a randomized, double-blind clinical trial was performed on 45 healthy elderly males (61–72 years-old), treated for 12 weeks with placebo (represented by medium-chain triglycerides), sardine-oil (abundant in n-3 polyunsaturated fatty acids-PUFAs- incorporated in triglycerides) or krill oil (abundant in n-3 PUFAs incorporated in phosphatidylcholine). The results of this study indicated that during the working memory task, both in the sardine and in the krill group the oxyhemoglobin concentrations in the cerebral cortex were significantly increased compared to the placebo group. In the calculation task, only the krill oil evoked an increase in oxyhemoglobin significantly different from the placebo group. Only the krill group showed a significantly lower differential value for P300 latency when compared to the placebo group [144,145].

### 9.2. Depression

The effect of krill oil supplementation was evaluated on cognition and depression-like behaviors both in pre-clinical and clinical studies.

One of the first studies in this field was carried out for seven weeks in rats, which received krill oil 0.2 g/rat/day, or imipramine 20 mg/kg/day (used as an anti-depressant reference drug) or placebo. At the end of the treatment, the cognitive abilities were tested by the Aversive Light Stimulus Avoidance Test (ALSAT) while the potential anti-depressant effect was tested by the Unavoidable Aversive Light Stimulus (UALST) and the Forced Swimming Test (FST). The results showed that krill oil treated rats had a significant ability to discriminate between the active and the inactive levers in the ALSAT test since the first day of training. Moreover, rats treated with krill oil and impramine exhibited significant improvement in behavior features such as lower levels in the UALST test since day three, and shorter immobility time in the FST test. The investigation also involved the expression of brain-derived neurotrophic factor (Bdnf) which was found to be upregulated in the hippocampus of krill oil treated rats [146].

Similar results were obtained by Zadeh-Ardabili and colleagues on mice treated with fish oil, krill oil, vitamineB12, imipramine or saline 5 mL/kg once per day for 14 days starting after one week of the Chronic Unpredictable Stress (CUS) paradigm overnight procedure. During CUS procedures, light was used to stress the mice overnight with an illumination of 10 W LED, 15 Hz for 12 hours for 3 weeks. The potential antidepressant effect of the treatments was tested by tail suspension test (TST) and FST. After animal sacrifice, the presence of oxidation markers was evaluated in the brain tissue. Both fish oil and krill oil significantly reduced the immobility factors and increased the time of climbing and swimming, similar to imipramine, when compared with the control group. Both the fish oil and the krill oil led to decreased malondialdehyde and hydrogen peroxide levels, decreased catalase activity, increased glutathione peroxidase levels and increased superoxide dismutase activities and glutathione levels in hippocampal tissue [147].

Another pre-clinical study, by Mendoza et al., investigated the role of krill oil on restraint stress in mice after reduced mobility. After two weeks of acclimation and handling, mice were immobilized for three weeks followed by a week dedicated to behavioral tests. During the four weeks of the study, the mice orally received PBS or cotinine (a nicotine-derivative) at 5 mg/kg, or cotinine plus krill oil 143 mg/kg. The results showed that cotinine alone reduced both the loss in cerebral synaptic density, memory deficits, anxiety and depression-like behaviors, but that the co-administration of cotinine plus krill oil was more effective than cotinine alone in reducing depression-like behaviors linked with reduced mobility. This confirms krill oil plays a role in depression mechanisms [148].

These encouraging pre-clinical data encouraged van der Wurff and colleagues to design and carry out a year long randomized, controlled, double-blind clinical trial on the effects of a krill oil supplementation on adolescent behaviors linked to learning, cognition, visual processing and mental well-being. The study included 264 adolescents between the ages of 13 and 15 years, who were divided in two cohorts. Cohort I started with 400 mg/day of EPA + DHA or placebo, and after three months the dose was increased to 800 mg of EPA + DHA per day. Cohort II started with 800 mg of EPA+DHA per day. The effects of these treatments were assessed by Omega-3 Index finger-prick blood measurements, Centre for Epidemiologic Studies Depression Scale evaluation and the Rosenberg Self Esteem questionnaire. The authors concluded that there was no evidence of an effect of krill oil in reducing depressive feelings or in inducing higher self-esteem. However, the authors reported that the results were affected by low adherence and drop-out, and for these reasons they suggest caution when interpreting the data [149,150].

## 10. Cancer

Only a few studies carried out with respect to krill oil and cancer cell lines are available. In a screening study carried out on several cancer cell lines such as cells derived from histiocytic lymphoma (U937), leukemia (K562, HL60), human hepatocarcinoma (SMMC-7721), bone metastasis of pancreatic cancer (PC3) and breast cancer (MDA-MB-231, MCF-7), a general inhibition of cancer cells proliferation by krill oil was observed [151].

In a comparison with fish oil, EPA and DHA incubated in human osteosarcoma cells for 24, 48 and 72 h, found only krill oil induced a significant inhibition of cancer cells proliferation (23, 50 and 64% of inhibition, respectively). Fish oil did not change the proliferation observed in control cells except for an increase observed after 24 h. On the contrary, EPA and DHA promoted cell proliferation and cell migration [152].

Some researchers have focused their attention on human colorectal cancer cells, in particular, Jayathilake and co-workers treated HCT-15, SW-480 and Caco-2 cells for 48 h with free fatty acid (FFA) extract from krill oil and fish oil. Their results indicated that krill oil and fish oil extracts inhibited cell proliferation in a similar manner but only an increase in mitochondrial membrane potential and consequent cell apoptosis was only observed with krill oil [153]. The same research group further investigated the antiproliferative effect of free fatty acid extract (FFAE) of krill oil on other human (DLD-1, HT-29 and LIM-2405) and murine (CT-26) colorectal cancer cells, in comparison with EPA, DHA (after 24 and 48 h) and oxaliplatin (after 24 h). Osteosarcoma, with colorectal cancer FFAE of krill oil, EPA and DHE inhibited cell proliferation and ROS formation in a similar way to oxaliplatin. FFAE of krill oil also induced a significant increase in caspase 3 and 9 levels which are markers of apoptosis [154]. An investigation of potential mechanisms of action was then carried out on DLD-1 and HT-29 cell lines treated with FFAE of krill oil at 8 and 24 h. From the evaluation of epidermal growth factor receptor (EGFR) signaling, the results indicated that FFAE of krill oil, at 0.03 and 0.12 µL/100 µL, induced reduction in EGFR, pEGFR, pERK1/2 and pAKT expression without any changes in total ERK1/2 and AKT levels. The expression of the ligand PD-L1 was significantly inhibited by FFAE of krill oil [155].

## 11. Exercise Performance

Krill oil has been associated with improvement of exercise and antioxidant/anti-inflammatory markers and several clinical trials have been carried out. The first was a small double-blind study carried out on 17 members of the Polish National Rowing Team. The rowers were divided in two groups: one received 1 g/day of krill oil for six weeks and one received placebo. The parameter of athletes was tested before, after 1 min and after 24 h, the latter of which was deemed maximum effort whereby participants had rowed 2000 m. Exercise induced an increase in erythrocytes or serum levels of some markers collected from rowers, such as superoxide dismutase, TNF-α and thiobarbituric acid reactive substances (TBARS, a marker of lipid peroxidation). While the other parameters did not differ from control and krill oil supplemented group, during recovery time TBARS continued to increase in the control group while the krill oil supplemented group showed significantly lower levels of lipid peroxidation. This suggests that krill oil could reduce the effort-associated free radical mediated injuries [156].

The effects of krill oil were also investigated to see its ability to influence exercise performance and post-effort immune function. In a small randomized clinical trial, 37 young (25.8 ± 5.3 years) athletes were divided into two groups: one received 2 g/day of krill oil for six weeks and the other received a placebo. A cycling time test was performed before and at the end of the supplementation period, where blood samples were collected before supplementation and immediately after exercise, or after 1 or 3 h, or at rest. The results showed that after six weeks of supplementation, the levels of peripheral blood mononuclear cell IL-2 production and natural killer cell cytotoxic activity 3 h post-exercise were significantly increased in krill oil supplemented athletes [157]. On this basis, other authors investigated the ability of krill oil to increase the body mass and the potential mechanism action behind this effect. To investigate the mechanism of action, they used C2C12 rat myoblasts (skeletal muscle) treated with krill oil or phosphatidylcholine derived from soy or control and observed that only krill oil was able to stimulate the mTOR pathway. In the clinical part of the study, a double-blind, placebo-controlled clinical trial was performed on resistance trained athletes receiving 3 g/day of krill oil or placebo during the resistance training program of eight weeks. At the end of the study, no difference in comprehensive metabolic panel, complete blood count or urine analysis were recorded between the two groups. However, krill oil was able to induce a significant increase in the lean body mass from baseline of about 2.1% [158].

Moreover, a particular mixed formulation, named ESPO-572^®^ composed of 75% PCSO-524^®^, which is green-lipped mussel oil and 25% krill oil, was effective in mitigation of exercise-induced muscle damage and cytokine-induced tissue degradation when administered for 26-day, 600 mg/day, in untrained men who underwent a running test [159]. As the levels of choline are known to maintain muscle function and exercise performance, a decrease in choline levels were recorded after high-resistance or high-intensity exercises. Storsve and colleagues performed a clinical trial to evaluate a possible protective effect by krill oil on this loss of choline. There were 47 triathletes placed randomly in two groups, one receiving 4 g/day of a particular formulation of krill oil named SuperbaBoost^TM^ for five weeks before the race and another group receiving placebo. Blood samples were collected pre-, immediately post-race and one day after the race and the serum choline and the choline metabolites were evaluated. As expected, the choline levels significantly decreased after the race, but significantly higher choline levels were found in athletes of the krill oil group compared with athletes who received placebo. These results seem to suggest that a krill oil supplement could prevent choline levels from falling and could avoid impairment in exercise performance, especially during high-resistance efforts [160].

## 12. Discussion and Future Perspectives

Considering the increased market of n-3 PUFA containing dietary supplements, supported by increased clinical evidence, there is a constant search for new n-3 PUFA sources and formulations [13].

Krill oil possesses several health benefits in clinical practice, in particular in cardiovascular disease risk factor management and in neurological diseases and inflammation [161]. It is commercialized in both the nutraceutical and pharmaceutical market in different dosage forms including soft gels, gummies, capsules and tablets.

However, despite many activities and functionalities that have been attributed to krill oil, the molecular pathways of actions are still in part unclear because few studies of pharmacodynamic are available and few have provided detailed information about molecular mechanisms of krill components such as astaxanthin, vitamin A, tocopherols, flavonoids, and minerals [162]. Most published RCTs do not provide any information regarding krill oil composition (except for the EPA and DHA content) [93]. In this regard, further studies are necessary to emphasize the relationship between krill oil components, mechanisms of action, health benefits and diversifying the different composition of krill oils for specific applications.

The importance of knowing the actions of the active components of krill oil is fundamental for the future development of new extraction techniques which could give rise to new chemical extract compositions for certain pathological conditions. To date, solvent and non-solvent extraction, super and subcritical fluid extraction and enzyme-assisted pre-treatment extraction represent the main technologies used for krill oil extraction, each of which have both advantages and disadvantages [163].

Among the active ingredients contained in krill oil, EPA and DHA constitute the main title of the products studied in clinical trials. EPA and DHA from krill oil are attached to phospholipids and to phosphatidylcholine. This composition promotes the efficiency of absorption of fatty acids into the blood when compared with omega-3 from fish oil [4]. However, the minor components contained in krill oil such as astaxanthin, alpha-tocopherol, vitamin A and flavonoids could exert pleiotropic activities and improve the bioaccessibility of EPA and DHA, even if data need to be clarified. Many studies on krill rarely detail the concentration in minor components, so it is hard to estimate their contribution to the final observed effects.

Krill oil products can be associated with other nutritional supplements to provide more benefits. Alvarez-Ricartes et al. demonstrated the efficacy of krill oil in addition to cotinine in the treatment of depressive symptoms in posttraumatic stress disorder people [164]. A study by Costanzo et al. found an association of krill oil with *Lactobacillus reuteri*, and vitamin D showed to reduce gut inflammation, reducing gut dysbiosis as well as increasing the epithelial restitution [135].

Currently, supplementation with krill oil is considered safe and well tolerated. Side effects are minimal or absent, and may include bloating, diarrhea and flatulence [165]. However, the available evidence is limited and further long-term RCTs, including many people, are needed to confirm both safety and efficacy of this nutraceutical. In addition, a cost/benefit analysis is necessary to better understand the implication of krill oil supplementation on health.

In conclusion, preliminary clinical data suggest that krill oil represent a valid supplement in the treatment of several conditions including CVDs, osteoarthritis, premenstrual syndrome and dysmenorrhea. Innovative technologies applied to improve krill oil purification and concentration could improve its cost-efficacy ratio.

## Figures and Tables

**Table 1:** The different content of polar lipids, monoacylglycerols, diacylglycerols, sterols, free fatty acids and triacylglycerols that have been found in different krill samples expressed as percent of total lipids.

Krill Sample	Polar Lipids	Monoacylglycerols	Diacylglycerols	Sterols	Free Fatty Acids	Triacyl Glycerols	Ref.
*Euphausia superba* in South Georgia	41.25	1.4	0.43	16.17	14.36	21.50	[19]
*Euphausia superba* in Gerlache Strait	44	0.9	3.6	1.4	8.5	40.4	[16]
*Euphausia superba* in Scotia Sea	45.7	0.4	1.3	1.7	16.1	33.3	[27]
*Euphausia superba* US AMLR Elephant Islands	ND	66–72	ND	4–6	1.1–1.8	22–38.4	[16]
*Euphausia superba* US AMLR Elephant Islands Extracted with ethanol	69.8	ND	ND	1.1	28.5	0.6	[28]
*Euphausia superba* US AMLR Elephant Islands Extracted with hexane	48.6	ND	ND	0.6	13.5	37.6	[29]
Krill oil from *Aker BioMarine*	80.7	ND	0.93	2.8	3.46	11.85	[30]

**Table 2:** The table reports the fractions expressed as % of total fatty acid characterized in krill samples.

Fatty Acid	Krill
C14:0 Myristic acid	5.0–13.0
C16:0 Palmitic acid	17.0–24.6
C16:1 (n-7) Palmitoleic acid	2.5–9.0
C18:1 (n-7) Cis-11-octadecenoic acid	4.7–8.1
C18:1 (n-9) Elaidic acid	6.0–14.5
C18:2 (n-6) Linoleic acid	ND–3.0
C18:3 (n-3) Alpha linolenic acid	0.1–4.7
C20:5 (n-3) Eicosapentaenoic acid	14.3–28.0
C22:5 (n-3) Docosapentaenoic Acid	ND–0.07
C22:6 (n-3) Docosahexaenoic acid (DHA)	7.1–15.7

**Table 3:** Experimental studies in which krill supplementation has been tested. Primary endpoints have been reported, as well as design and duration of the studies. (TG = triglycerides).

Classification of Health Benefits	Model	Diets	Duration of Supplementation	Outcomes	References
**Metabolic disorders**	Sprague Dawley rats HFD	100 and 200 g/krill oil (KO)	2 weeks	↓ serum lipid levels	[104]
	HFD mice	1.25–2.5–5% KO	8 weeks	↓ liver TGs, cholesterol and serum cholesterol and glucose	[105]
	hTNFα over-expressing mice	Krill powder (4.3% of proteins)	8 weeks	↓ liver and plasma TGs, hepatic expression SREBP2, ↑ β-oxidation, ↓ inflammation	[106]
	LDLR-KO mice fed with a western diet + Pacific krill	8-HEPE (100 mg/kg)	18 weeks	↓ plasma LDL and total cholesterol, ↑ HDL, ↓ hepatic TG levels	[107]
	Dyslipidemic and diabetic non-human primates	150 mg/Kg/day		↓ plasma total and LDL-cholesterol, and TGs, ↑ HDL-cholesterol	[108]
	C57BL/6J mice fed with HFD	5% krill powder	12 weeks	↓ body weight gain, the fat accumulation in tissue adipose and liver, ↓ serum LDL, ↑ glucose tolerance. ↓ oxidative damage in liver	[114]
	Rats HFD	2.5% krill	12 weeks	↓ body weight gain	[109]
	Obesity model in castrated New Zealand white rabbits	600 mg/day	8 weeks	↑ insulin sensitivity and secretion, ↓ fasting blood glucose	[110]
	HFD combined with thermoneutral animal housing	KO (containing EPA ~13%, DHA ~8%)	24 weeks	↓ liver steatosis	[152]
	Randomized controlled study on 36 individuals	4 g/day	8 weeks	↑ EPA, DHA and DPA in krill group	[111]
	Human with borderline or high TG levels	0.5, 1, 2, or 4 g/day for	6 and 12 weeks	↓ plasma TGs	[112]
	Randomized cross-over clinical trial on 25 moderately hyperTGmic subjects	1000 mg/day	4 weeks	↑ plasma HDL and apolipoprotein AI levels	[113]
	11 obese men	4 g/day per os	24 weeks	↓ anthropometric parameters and blood AEA and 2-AG	[122]
	63 obese subjects	2 g/day	4 weeks	↓ 2-AG levels, no significant effect on antropometric	[123]
**Pretection** **against myocardial infarct**	MI and euthanasia after 7 days	KO containing 0.47 g/100 g EPA + DHA	14 days of pretreatment with KO before MI	↓ heart and lung hypertrophy, and and inflammation	[118]
**Vascular function**	34 participants with type 2 diabetes	1 g/day in PUFA	4 weeks	↑ endothelial function ↓ blood C peptide levels and HOMA scores	[119]
**Gut microbiota and IBD**	ICR mice fed with HFD	fish oil (600 μg/g/day), KO (600 μg/g/day) and their mixture (300 + 300 μg/g/day	12 weeks	↓ obesity, ↑ positive *phyla* (i.e., *Bacterioides* and *Lactobacilli)*	[124]
	Obesity and hyperlipidemia induced by HFD+ high sugar diet	100, 200, 600µg/g/day	12 weeks	↑ microbiotic alteration and cardiometabolic parameters	[125]
		100, 200 and 600 mg/kg	7 weeks	↑ abundance of *Lactobacillus* spp. and short-chain fatty acids producers	[126]
	Dextran sulfate sodium (DSS)-induced colitis in mice	mixture of KO, *Lactobacillus reuteri* and vitamin	4 weeks	↑ clinical and histological scores, restore epithelial restitution, ↓ proinflammatory cytokines	[110]
	DSS-induced colitis in mice	5%	4 weeks	↓ disease activity index and TNF-α and IL-1β levels	[128]
	DSS-induced colitis in mice	KO-entrapped liposomes (containing 42% w/w phospholipids, ≥26.5 w/w% total Omega-3, ≥8.5% w/w DHA, ≥14.5% w/w EPA, and 0.125±0.025 w/w% astaxanthin)	8 weeks	↓TNF and IL6 and the systemic levels of endotoxin, ↑ hydrophobic protective barrier	[129]
	*C. rodentium* infected mice	1.5 g KO	4 weeks	↓ inflammatory pathway, ↓of *Rickettsiales* and several species of *Lactobacillus*	[130]
**Arthritic disease**	Mice experimental models of inflammatory arthritis	KO diet, in which EPA + DHA were 0.44 g/100 g of KO diet	2 months	↓ infiltration of inflammatory cells and hyperplasia at synovial layer	[132]
	hTNF-α over-expressing mice		6 weeks	↓ markers of fatty acid oxidation	[133]
	Randomized, double blind, placebo controlled clinical trials on 90 patients with CVD and/or osteoarthritis and/or rheumatoid arthritis	300 mg/day		↓ CRP levels and pain (about 29%), stiffness (about 20%) and functional impairment (about 23%).	[134]
	Randomized, double-blind, parallel-group, placebo-controlled study, 50 patients with mild knee pain	2 g/day	30 days	↓ knee pain, ↑ motion of both the knees	[135]
	260 Australian patients affected by knee OA	2 g/day	6 months (ongoing)	↑ knee pain and in size of knee synovitis/effusion	[11]
**Neurodegeneration**	Human neuronal SH-SY5Y cell line			↓ oxidative stress and mitochondrial protection	[137]
	Aged rats	20, 50 mg/kg/day	7 days	↑ cholinergic trasmission, muscarinic receptors and choline transporters	[139]
	LPS -induced mice model of Alzheimer	80 mg/kg/day	4 weeks	↓ iNOS, COX-2, NFkB, ROS and malondialdehyde levels, amyloid beta (1–42) peptide	[141]
	Senescence-accelerated prone mouse strain 8 (SAMP8)	1% of KO	12 weeks	↑ cognitive function and the anxiety, ↓ memory deficit and learning, ↓ β-amyloid Aβ42 accumulation	[142]
	Amyloid Aβ25-35-induced mouse model of Alzheimer	100, 200 or 500 mg/Kg/day	14 days	↓ latency in the Morris water maze test, ↓ Bax/Bcl-2 ratio in the brain and ↓ levels of ROS, malondialdehyde and NO	[143]
	Randomized, double-blind clinical trial on 45 healthy elderly males (61–72 years-old)	sardine-oil, KO or placebo	12 weeks	↑ cognitive capacity	[144]
		KO 0.2 g/rat/day, or imipramine 20 mg/kg/day	7 weeks	↑ cognitive abilities, in behaviour features and Bdnf	[145]
		KO or vitamine B12 or imipramine or saline 5 mL/kg	14 days	↓ malondialdehyde and hydrogen peroxide levels, catalase activity, ↑ glutathione peroxidase levels, superoxide dismutase activities and glutathione levels	[146]
	Immobility-induced murine depression model	PBS, or cotinine (a nicotine-derivative) 5 mg/kg, or cotinine plus KO 143 mg/kg	4 weeks	↓ depression-like behaviours	[147]
	Randomized, controlled, double-blind clinical trial on 264 adolescent (13–15 years)	cohort I: 400 mg/day of EPA + DHA or placebo, and after 3 months increased the dose to 800 mg/day of EPA + DHA. cohort II: 800 mg/day of EPA + DHA	1 year	no evidence about an effect on depressive feelings, low adherence	[148,149]
**Cancer**	Several human and murine colorectal cancer cells	0.03 and 0.12 µL/100 µL	24–48 h	↓ cell proliferation, ↓ expression of EGFR, pEGFR, pERK1/2 and pAKT	[154]
**Exercise performance**	Double-blind on 17 rowers members of the Polish National Rowing Team	1 g/day of KO	6 weeks	↑ erythrocytes or serum levels of superoxide dismutase, TNF-α and thiobarbituric acid	[155]
	Randomized clinical trial on 37 young athletes	2 g/day of KO	6 weeks	↑ levels of peripheral blood mononuclear cell IL-2 production and natural killer cell cytotoxic activity, 3 h post-exercise	[156]
	Double-blind, placebo-controlled clinical trial	3 g/day of KO or placebo during the resistance training	8 weeks	↑ in the lean body mass (about 2.1% vs. baseline)	[157]
		ESPO-572^®^ (75% of PCSO-524^®^ and 25% KO) 600 mg/day	26 days	↑ mitigation of exercise-induced muscle damage and cytokine-induced tissue degradation	[158]
	47 triathletes randomized supplemented before the race.	4 g/day of a KO (Superba Boost^TM^)	5 weeks	↑ exercise performance, especially during high-resistance efforts	[159]

## Data Availability

Not applicable.
